# Extremes of Fetal Fraction on Noninvasive Prenatal Screening and Placental Histopathology: Is There an Association?

**DOI:** 10.3390/jcm14228185

**Published:** 2025-11-18

**Authors:** Zachary D. Stanley, Sherri Besmer, Leah Hong, Megan Zierold, Erin Fey, Regina Huang, Carole Vogler, Jessenia Guerrero, Niraj R. Chavan

**Affiliations:** 1 Division of Maternal-Fetal Medicine, Saint Louis University School of Medicine (SLU SOM), 6420 Clayton Rd, Saint Louis, MO 63117, USA; 2 Division of Anatomic and Clinical Pathology, SLU SOM, St. Louis, MO 63104, USA; 3 Henry Ford Health, Maternal-Fetal Medicine, Detroit, MI 48202, USA; 4 School of Medicine, Saint Louis University, St. Louis, MO 63104, USA; 5 SSM Health, Division of Prenatal Genetics, St. Louis, MO 63117, USA; erin.fey@slucare.ssmhealth.com; 6 Advanced Health Data Institute, SLU SOM, St. Louis, MO 63104, USA

**Keywords:** fetal fraction, noninvasive prenatal genetic screening, placental/perinatal pathology

## Abstract

**Objective**: To evaluate the association between low and high fetal fraction (FF) of cell-free fetal DNA on non-invasive prenatal screening (NIPS) and placental pathology. **Methods**: We undertook a prospective cohort study of patients undergoing NIPS between July 2022 and July 2023 through Natera Inc. Based on the FF percentile, the study cohort was divided into three groups: high FF (≥95th%), low FF (≤5th%), and a control group (FF 6th–94th%). Our primary study outcome was a composite of high-risk placental lesions. We compared the occurrence of the primary study outcome across the study groups using the chi^2^ test. Multivariable regression analyses were performed to predict the likelihood of the primary outcome based on the FF percentile. Selected obstetric and neonatal outcomes were assessed as secondary outcomes. **Results**: The primary outcome was present in 11 (50.0%), 19 (48.7%), and 11 (35.5%) of participants in the low FF, high FF, and control cohorts, respectively (*p* = 0.46). In an adjusted model, the FF percentile was not associated with the primary outcome (aOR 2.41 (0.72–8.42) for low FF, aOR 1.55 (0.51–4.82) for high FF). Chorangiosis (*p* = 0.02) and fetal inflammatory response (*p* = 0.002) were seen more commonly in the low and high FF groups. Spontaneous preterm birth was more common in the low FF group (*p* = 0.04). **Conclusions**: Our study did not identify a correlation between high-risk histopathological patterns and extremely low or high FF when compared to a control cohort. Chorangiosis and fetal inflammatory response were found more commonly in the low and high FF groups.

## 1. Introduction

Noninvasive prenatal screening (NIPS) is an increasingly common genetic screening modality in routine obstetric practice. NIPS uses fetal cell-free DNA (cfDNA) to screen for common aneuploidies with a high degree of sensitivity and specificity. The test’s accuracy depends on fetal fraction, the percentage of fetal cfDNA in maternal circulation, which is reported as a quality control measure [1].

The term fetal fraction and fetal cfDNA is a misnomer, as the DNA fragments are placental in origin. Throughout pregnancy, the villous trophoblast of the placenta undergoes continuous turnover, releasing apoptotic debris and cfDNA into maternal circulation [2,3]. Because cfDNA is derived from the placenta, it has been hypothesized that it may be an indicator of placental integrity [4].

Previous studies have correlated high and low fetal fractions with adverse pregnancy outcomes [5,6]. Low fetal fraction has been associated with hypertensive disorders of pregnancy, gestational diabetes mellitus, and other outcomes associated with placental compromise [1,4,7,8,9,10,11]. High fetal fraction has been associated with fetal growth restriction, spontaneous preterm birth, and hypertensive disorders of pregnancy [6,12,13,14]. These studies have largely focused on adverse obstetric and neonatal outcomes, many of which are often used as surrogates for placental dysfunction [7,15,16]. One prior study evaluated placental pathology in pregnancies with low and high fetal fractions on NIPS, but did so retrospectively on placentas that were sent per the delivering clinician’s preference. Their study showed an association with low fetal fraction and markers of chronic inflammation in the placenta [17].

The objective of this study was to interrogate the relationship between extreme levels of cfDNA on NIPS and findings on placental pathology. Because fetal cfDNA originates from placental trophoblastic cells, we hypothesized that extremes of fetal fractions of cfDNA may herald abnormal placental development as evidenced by the occurrence of high-risk placental lesions on histopathological analysis.

## 2. Materials and Methods

We conducted a prospective cohort study of all pregnant individuals undergoing cfDNA aneuploidy screening at our institution. Our practice is to offer universal cfDNA aneuploidy screening to all patients, regardless of their a priori risk for aneuploidy. Participants were identified in collaboration with Natera, Inc. (San Carlos, CA, USA) if they underwent cfDNA aneuploidy screening at one of our clinic locations from July 2022 to July 2023. Potential participants were identified if the fetal fraction, based on the patient’s weight and gestational age at the time of testing, was categorized as either low or high. Low fetal fraction was defined as a fetal fraction less than or equal to the 5th percentile, and high fetal fraction was defined as greater than or equal to the 95th percentile. The 5th and 95th percentiles were selected as the extremes of fetal fraction, in congruence with previous studies in this field [13].

Pregnant individuals were approached and informed consent was obtained for study inclusion if they had a low-risk result on NIPS, were English-speaking, had a singleton pregnancy, were not prescribed anticoagulation, and planned to deliver at our institution. They were excluded if they did not meet the inclusion criteria, delivered prior to consenting to study participation, or if there was a known or highly suspected chromosomal abnormality. A control cohort was created by approaching patients who were not identified as either high or low fetal fraction (i.e., with a fetal fraction between the 6th and 94th percentile) after matching their age, weight, and gestational age at the time of NIPS to the low and high fetal fraction participants. These variables were chosen given their potential for impacting the fetal fraction [18,19,20].

A comprehensive histopathologic checklist was created in collaboration with three perinatal pathologists at our institution who are both fellowship-trained and experienced with placental/perinatal pathology. The placental lesions captured by the checklist included gross cord abnormalities, acute chorioamnionitis/amniotic fluid infection sequence, chronic inflammatory lesions, fetal vascular malperfusion/fetal thrombotic vasculopathy, maternal vascular malperfusion, and intervillous lesions (Appendix A Table A1). These lesions were included in the checklist due to their reported associations with adverse pregnancy outcomes [21]. At the time of delivery, each placenta was sent to the pathology laboratory for processing. The placental specimens then underwent a systematic, blinded review using the abovementioned checklist.

## 3. Study Outcomes

The primary study outcome was structured as a composite of placental findings known to be most strongly associated with adverse pregnancy outcomes, including stillbirth, spontaneous preterm birth, fetal growth restriction, and central nervous system injury at term [21]. Additional findings that were noted by the reviewing pathologists were recorded either in a “miscellaneous” category checklist (which included chorangiosis, chorangiomatosis, meconium effect, villous immaturity, and villous edema) or “other” category by free text.

The secondary study outcomes included selected pregnancy and neonatal outcomes, which were abstracted from the electronic medical record for each study participant and stored in RedCap [22]. The obstetric outcomes included hypertensive disorders of pregnancy, fetal growth restriction, placental abruption, oligohydramnios, spontaneous preterm birth, and postpartum hemorrhage. Hypertensive disorders of pregnancy were classified based on the 2020 American College of Obstetricians and Gynecologists consensus guidelines [23]. Fetal growth restriction was defined as an estimated fetal weight or abdominal circumference less than the tenth percentile for gestational age [24]. Abruption was defined clinically by the delivering clinician. Oligohydramnios was defined as a deepest vertical pocket measurement of less than two centimeters on transabdominal ultrasound. Postpartum hemorrhage was defined as a quantitative blood loss documented as greater than or equal to one liter, regardless of mode of delivery. The neonatal outcomes included birthweight, small for gestational age (SGA), large for gestational age (LGA), poor Apgar scores at birth, need for NICU admission, morbidity of prematurity, and stillbirth or neonatal death. SGA and LGA status were determined based on Olsen percentiles and calculated using PediTools [25,26]. Poor Apgar scores at birth were defined as less than five at one minute or less than seven at five minutes. Morbidity of prematurity included any of the following: respiratory distress syndrome, intraventricular hemorrhage, necrotizing enterocolitis, bronchopulmonary dysplasia, or retinopathy of prematurity.

Demographic and clinical characteristics as well as maternal and neonatal clinical outcomes were compared across the three study groups—i.e., patients with high FF (≥95th percentile), low FF (≤5th percentile) and those in the control group (FF between the 6th and 94th percentile) using one-way Analysis of Variance (ANOVA) or the Kruskal–Wallis test for continuous variables (depending on normality of distribution) and chi squared or Fisher’s exact test for categorical variables, as applicable. Similarly, the occurrence of each of the prespecified histopathology findings, as well as the primary study outcome overall, was compared across the three study groups using the chi-squared or Fisher’s exact test, as appropriate. Multivariable regression analyses were undertaken to predict the likelihood of identifying the primary study outcome (high-risk placental lesions) based on the fetal fraction percentile, while adjusting for maternal age, BMI, gestational age at time of NIPS, self-identified race, nulliparity, tobacco use, and chronic hypertension. A similar regression was performed for selected obstetric and neonatal outcomes, including hypertensive disorders of pregnancy, gestational diabetes, spontaneous preterm birth, fetal growth restriction, and NICU admission. Statistical significance was set at *p* ≤ 0.05. All analyses were performed using RStudio (version 2023).

## 4. Results

**Figure 1:**
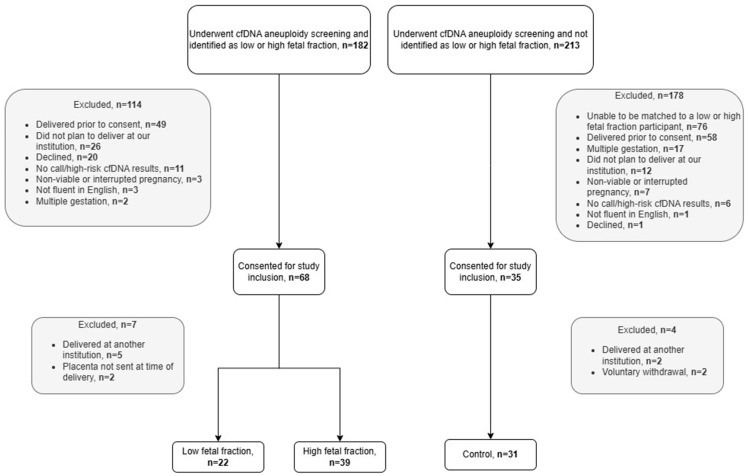
Flow chart defining study cohort.

Out of the 182 pregnant individuals who underwent NIPS and were identified as having low or high fetal fraction during the study period, 68 individuals were consented, including 22 in the low fetal fraction group and 39 in the high fetal fraction group. An additional 213 pregnant individuals underwent NIPS during the study period and were considered for inclusion in the control cohort. In total, 35 individuals consented to study participation after matching. Reasons for exclusions and attrition in these groups are outlined in Figure 1.

Demographic and clinical characteristics across the three study groups are represented in Table 1. There were no statistically significant differences noted in the age or gestational age at the time of NIPS across all groups (Table 1). Our study population predominantly self-identified as non-Hispanic black (67.4%), with the distributions of self-reported race otherwise similar across all groups. The median BMI was 25.9 kg/m^2^ in the low fetal fraction group and 29.8 kg/m^2^ in the high fetal fraction group (*p* = 0.01). Of note, the reported median fetal fraction (percent) for the low, high, and control cohorts was 4.2%, 16.6%, and 7.6%, respectively (*p* < 0.001). Tobacco use was significantly different amongst the groups, with 13.6%, 33.3%, and 6.5% of the low, high, and control groups reporting current use during the current pregnancy (*p* = 0.02). There were no other significant differences in any of the clinical characteristics under consideration.

Data pertaining to the primary study outcome, including each of the histopathology findings that contributed towards the composite, are presented in Table 2. The primary study outcome was noted in 50% (*n* = 11/22), 48.7% (*n* = 19/39), and 35.5% (*n* = 11/31) of placentas from the low, high, and control groups, respectively (Table 2). When compared to the control group, there were more findings consistent with a fetal inflammatory response in the low (*n* = 6, 27.3%) and high (*n* = 9, 23.1%) fetal fraction groups. There was also a higher incidence of chorangiosis in the low (*n* = 3, 13.6%) and high (*n* = 7, 17.9%) fetal fraction groups, as compared to no such occurrences noted in the control group.

A total of 57 (62%) placentas had “additional” findings that were not captured by the placental pathology checklist but were commented upon by the reviewing pathologist. Of those, 11 (50%) were in the low fetal fraction group, 29 (74%) were in the high fetal fraction group, and 17 (54%) were in the control group. The most common “additional” findings were subchorionic fibrin thrombus/plaque (*n* = 18), perivillous fibrin thrombus/deposition (*n* = 15), intervillous thrombus (*n* = 9), and squamous metaplasia (*n* = 6). The occurrence of other histopathological findings from the comprehensive checklist was not noted to differ significantly across the three study groups (Appendix A Table A1).

Secondary study outcomes, including maternal and neonatal outcomes, are presented in Table 3. Significant differences were noted in the mode of delivery as well as the rate of spontaneous preterm birth (sPTB) across the study groups, with the control group noted to have the highest cesarean delivery rate (*n* = 15, 48.4%) and the low fetal fraction group noted to have the highest rate of sPTB (*n* = 6, 27.3%). There were no other significant differences noted in any of the maternal and neonatal clinical outcomes across the study groups. The gestational age at the time of delivery was not statistically different between the groups and was approximately 39 weeks. The incidence of spontaneous preterm birth was highest in the low fetal fraction group, impacting 27.3% of those pregnancies, and this was statistically significant when compared to the high fetal fraction and control groups. The cesarean section rate did not differ significantly between the low and high fetal fraction groups, at 13.6% and 25.6% of births in each group, respectively. Of note, the cesarean section rate was significantly higher in the control group at 48.4%. None of the other selected obstetric or neonatal outcomes were statistically significant between the groups (Table 3).

**Figure 2:**
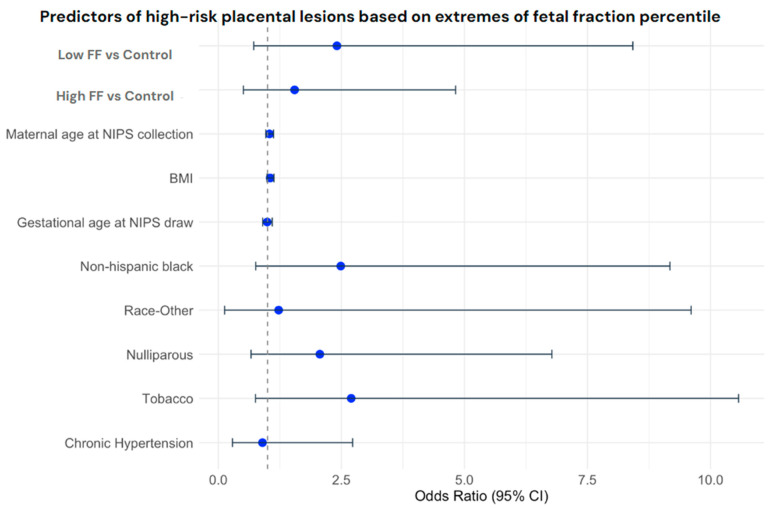
Forest plot showing the odds ratios and confidence intervals for variables included in the adjusted model for the primary placental pathology composite outcome.

After adjusting for variables known to influence fetal fraction, extremes of fetal fraction, including both low fetal fraction (aOR 2.41, CI 0.72–8.42) and high fetal fraction (aOR 1.55, CI 0.51–4.82), did not predict a statistically significant change (increase or decrease) in the likelihood of identifying the primary study outcome on histopathology as compared to those with a fetal fraction in the 6th–94th percentile range (control group) (Figure 2). No significant findings were found with regard to the secondary clinical outcomes in a similar adjusted model.

## 5. Discussion

We undertook a prospective cohort study to examine the relationship between extremes of fetal fraction on NIPS and the occurrence of high-risk placental lesions that have been shown to be associated with adverse perinatal outcomes. Prior studies have focused on assessing associations between fetal fraction and pregnancy outcomes that are thought to be mediated by the placenta. For instance, recent systematic reviews such as Chen et al. [10] and Sapantzoglou et al. [11] highlight consistent links between low fetal fraction and outcomes like hypertensive disorders, fetal growth restriction, and preterm birth, but rely on clinical outcomes as proxies for placental compromise. Our study adds histopathologic data to this literature by directly evaluating the placenta using a comprehensive, standardized approach.

Despite this detailed review, our prospective cohort study was unable to demonstrate a clear association with fetal fraction extremes and our high-risk placental histopathology composite variable, suggesting that the relationship between fetal fraction and pregnancy risk may not be explained by a uniform or easily detectable pattern of structural placental anomalies. Two interesting associations were identified, however: chorangiosis and fetal inflammatory response. Chorangiosis was seen more often in our high and low fetal fraction cohorts compared to the control. Chorangiosis, characterized by an increased number of capillaries within terminal chorionic villi, is regarded as a histopathological marker of chronic placental hypoxia or hypoperfusion and is associated with adverse clinical outcomes [27,28]. Chorangiosis may be seen in the setting of diabetes, preeclampsia, or hypertension, or in association with cord lesions [27]. Fetal inflammatory response was also seen more commonly in the high and low fetal fraction groups when compared to control placentas. Fetal inflammatory response is characterized by inflammation of the fetal components of the placenta and is seen in ascending intrauterine infection. Fetal inflammatory response includes chorionic vasculitis, umbilical vasculitis, and funisitis. Fetal inflammatory response is associated with poor fetal outcomes [29]. The quantification of the stage and grade of the inflammatory responses, in addition to the relationships between the maternal and fetal inflammatory responses identified, were beyond the scope of this preliminary study, but may be a focus of interest for future studies.

The median BMI was significantly different between the high and low fetal fraction cohorts, which is consistent with prior studies that have shown that this variable is associated with differences in the fetal fraction [20]. Low fetal fraction was associated with spontaneous preterm birth in our cohort, which has not been a consistent finding in prior studies [11].

Our study has several strengths. All study participants were identified using the same laboratory and the same NIPS platform, potentially diminishing any variation in the measurement of fetal fraction. The prospective study design and comprehensive nature of our placental pathology checklist are unique strengths. One prior study by Suresh and colleagues evaluated abnormal fetal fraction and placental pathology with adverse pregnancy outcomes. This was a retrospective study and only included placental specimens that were sent at the time of delivery (per the delivering clinician’s preference). They also used the 25th and 75th percentiles as cut-offs, whereas we looked at more extreme values [17]. We considered all women who obtained cfDNA aneuploidy at our institution, and a majority of our study cohort identified as Black. Prior studies evaluating low and high fetal fractions have been in predominantly Caucasian populations.

We attempted to recruit a control cohort that was well-matched when considering variables that have been shown to influence fetal fraction. Our control cohort was limited, however, by our inability to match for all chronic medical conditions/comorbidities, including hypertensive disorders and substance use, as this could impact placental pathology findings independent of the fetal fraction. Since matching all such conditions and characteristics would pose challenges to study feasibility, our approach focused on accounting for these covariates as potential confounders in the multivariable regression analyses. The participants in our study all consented prior to delivery, and each medical record was thoroughly reviewed by a clinical member of the research team.

While this study was exploratory in nature, we recognize that a higher-powered study may be required to identify emerging histopathological patterns, in addition to elucidating more information about the associations with chorangiosis and fetal inflammatory response that were detected. Some placental lesions that portend high risk of adverse perinatal outcomes and high recurrence risks (e.g., massive histiocytic intervillositis and massive perivillous fibrin deposition) are very rare overall, with limited case series published [30].

Participant recruitment was complicated by the prospective study design and the limited time from notification of participant eligibility from Natera, Inc. to the time of delivery. Though we consider our study population to be diverse, participants were approached from a single institution, which may reduce the generalizability of our findings.

## 6. Conclusions

NIPS is a powerful tool for fetal aneuploidy screening. The fields of obstetrics and prenatal genetics continue to evolve, increasing the utilization of this technology to enhance the risk-stratification of pregnancies. Although this investigation did not elucidate clear placental pathology patterns in patients with extremely low or high fetal fractions, the previous literature has shown that the fetal fraction itself is a marker of pregnancy risk. This study underscores the need for future investigations involving larger sample sizes. Many of the increased risks that low or high fetal fractions predict are thought to be mediated by the placenta. As NIPS continues to become a more common practice for routine aneuploidy screening, we anticipate further uses beyond that of genetic screening for pregnancies, which will strengthen our ability to individualize patient care to improve maternal and neonatal outcomes.

## Figures and Tables

**Table 1:** Demographic and clinical characteristics of the entire study cohort.

	Low Fetal Fraction, *n* = 22	High Fetal Fraction, *n* = 39	Control, *n* = 31	*p* (High, Low, Control)
Maternal age at blood draw (years)	27.8 ± 8.8	28.5 ± 6.7	28.9 ± 6.6	0.85
Gestational age at blood draw (weeks)	11.6 (10.2, 14.2)	12.4 (11.8, 15.4)	13.0 (12, 13.7)	0.17
Fetal fraction (%)	4.2 (3.5, 4.8)	16.6 (12.1, 20.0)	7.6 (5.6, 10.0)	<0.001 *
BMI (kg/m^2^)	25.9 (23.4, 28.6)	29.8 (26.2, 37.8)	28.3 (25.5, 35.7)	0.02 *
Insurance				0.35
Private	8 (36.4)	10 (25.6)	5 (16.7)	
Public	14 (63.6)	28 (71.8)	25 (83.3)	
Self-reported race				0.15
Non-Hispanic Black	15 (68.2)	22 (56.4)	25 (80.6)	
Non-Hispanic White	5 (22.7)	15 (38.5)	4 (12.9)	
None of the above	2 (9.1)	2 (5.1)	2 (6.5)	
Nulliparous	10 (47.6)	12 (30.8)	8 (25.8)	0.24
Current tobacco use	3 (13.6)	13 (33.3)	2 (6.5)	0.02 *
Chronic hypertension	4 (18.2)	13 (33.3)	6 (19.4)	0.31
Diabetes mellitus				
Pregestational	1 (4.5)	1 (2.6)	2 (6.5)	0.82
Gestational	1 (4.5)	2 (5.1)	5 (16.1)	0.32

Data are presented as *n* (%) and either mean ± SD or median (IQR) based on the distribution of the data. BMI = body mass index. * Represents statistically significant differences at *p* ≤ 0.05.

**Table 2:** Primary placental composite outcome with the individual variables included in the composite.

	Low	High	Control	*p*
	*n* = 22	*n* = 39	*n* = 31	(Low, High, Control)
Composite outcome, *n* (%)	11 (50.0)	19 (48.7)	11 (35.5)	0.46
Chronic inflammatory lesions				
Chronic deciduitis with plasma cells, *n* (%)	2 (9.1)	5 (12.8)	2 (6.5)	0.75
CVUE, high-grade, *n* (%)	1 (4.5)	4 (10.3)	1 (3.2)	0.56
Fetal vascular malperfusion/fetal thrombotic vasculopathy				
High-grade, *n* (%)	1 (4.5)	1 (2.6)	0 (0.0)	0.71
Fetal thrombosis, *n* (%)	1 (4.5)	3 (7.7)	1 (3.2)	0.85
Villous avascularity/hypovascularity, *n* (%)	4 (18.2)	6 (15.4)	4 (12.9)	0.93
Villous stromal-vascular karyorrhexis, *n* (%)	2 (9.1)	4 (10.3)	0 (0.0)	0.16
Maternal vascular malperfusion				
Accelerated villous maturation, *n* (%)	2 (9.1)	2 (5.1)	1 (3.2)	0.72
Distal villous hypoplasia, *n* (%)	0 (0.0)	0 (0.0)	0 (0.0)	/
Villous infarcts, *n* (%)	0 (0.0)	2 (5.1)	0 (0.0)	0.5
Decidual arteriopathy/decidual vasculopathy/acute atherosis, *n* (%)	2 (9.1)	4 (10.3)	2 (6.5)	0.9
Retroplacental hematoma/hemorrhage, *n* (%)	2 (9.1)	0 (0.0)	0 (0.0)	0.06
Miscellaneous				
Villous immaturity, *n* (%)	0 (0.0)	0 (0.0)	2 (6.5)	0.17

**Table 3:** Obstetric and neonatal outcomes of the study cohort.

	Low Fetal Fraction, *n* = 22	High Fetal Fraction, *n* = 39	Control, *n* = 31	*p* (High, Low, Control)
Obstetric outcomes				
Gestational age at delivery (weeks)	38.7 (36.9, 39.3)	39.0 (38.2, 39.4)	38.9 (37.4, 39.2)	0.4
Mode of delivery				0.03 *
Vaginal	18 (81.8)	26 (66.7)	16 (51.6)	
Operative vaginal	1 (4.5)	3 (7.7)	0	
Cesarean section	3 (13.6)	10 (25.6)	15 (48.4)	
Hypertensive disorders of pregnancy	11 (50.0)	12 (30.8)	13 (41.9)	0.8
Fetal growth restriction	2 (9.1)	4 (10.3)	1 (3.2)	0.6
Abruption	0	1 (2.6)	0	1
Oligohydramnios	2 (9.1)	1 (2.6)	0	0.18
Spontaneous preterm birth (<37 weeks)	6 (27.3)	2 (5.1)	6 (19.4)	0.04 *
Postpartum hemorrhage	1 (4.5)	5 (12.8)	4 (12.9)	0.64
Neonatal outcomes				
Birthweight (g)	2870 ± 508	3142 ± 511	3096 ± 558	0.15
SGA	0	3 (7.7)	1 (3.2)	0.67
LGA	0	2 (5.1)	1 (3.2)	
NICU admission	7 (31.8)	7 (17.9)	11 (35.5)	0.22
Poor Apgar	1 (4.5)	3 (7.7)	3 (9.7)	0.89
Morbidity of Prematurity	0	0	1 (3.2)	0.58
Stillbirth or neonatal death	0	0	0	

Data are presented as n (%) and either mean ± SD or median (IQR) based on the distribution of the data. SGA = small for gestational age <10th percentile for birthweight, LGA = large for gestational age >90th percentile for birthweight, NICU = neonatal intensive care unit. Poor Apgar was defined as 1-min Apgar <5 or 5-min Apgar <7. Morbidity of prematurity included neonatal sepsis, intraventricular hemorrhage, necrotizing enterocolitis, bronchopulmonary dysplasia, and retinopathy of prematurity. * Represents statistically significant differences at *p* ≤ 0.05.

## Data Availability

The original contributions presented in this study are included in the article. Further inquiries can be directed to the corresponding author.

## Appendix A

**Table A1:** Histopathologic placental findings of the study cohort.

	Low Fetal Fraction, *n* = 22	High Fetal Fraction, *n* = 39	Control, *n* = 31	*p* (High, Low, Control)
Small placental disk	6 (27.3)	10 (26.3)	7 (22.6)	0.91
Large placental disk	1 (4.5)	3 (7.7)	3 (9.7)	0.89
Low fetal/placental ratio	1 (4.5)	5 (12.8)	2 (6.5)	0.58
High fetal/placental ratio	1 (4.5)	1 (2.6)	2 (6.5)	0.82
Gross cord abnormality				
Marginal insertion	3 (13.6)	2 (5.1)	2 (6.5)	0.53
Membranous/velamentous insertion	0	0	0	
Single umbilical artery	0	0	1 (3.2)	0.58
Hypercoiled	6 (27.3)	11 (28.2)	5 (16.1)	0.46
Hypocoiled	0	1 (2.6)	0	1
Amniotic fluid infection sequence				
Maternal inflammatory response	7 (31.8)	9 (23.1)	4 (12.9)	0.27
Fetal inflammatory response	6 (27.3)	9 (23.1)	0	0.002 *
Chronic inflammatory lesions				
Chronic deciduitis with plasma cells	2 (9.1)	5 (12.8)	2 (6.5)	0.75
CVUE, low-grade	1 (4.5)	3 (7.7)	3 (9.7)	0.89
CVUE, high-grade	1 (4.5)	4 (10.3)	1 (3.2)	0.56
Fetal vascular malperfusion/fetal thrombotic vasculopathy				
Low-grade	1 (4.5)	2 (5.1)	4 (12.9)	0.47
High-grade	1 (4.5)	1 (2.6)	0	0.71
Fetal thrombosis	1 (4.5)	3 (7.7)	1 (3.2)	0.85
Villous avascularity/hypovascularity	4 (18.2)	6 (15.4)	4 (12.9)	0.93
Villous stromal-vascular karyorrhexis	2 (9.1)	4 (10.3)	0	0.16
Maternal vascular malperfusion				
Accelerated villous maturation	2 (9.1)	2 (5.1)	1 (3.2)	0.72
Distal villous hypoplasia	0	0	0	
Villous infarcts	0	2 (5.1)	0	0.5
Decidual arteriopathy/decidual vasculopathy/acute atherosis	2 (9.1)	4 (10.3)	2 (6.5)	0.9
Retroplacental hematoma/hemorrhage	2 (9.1)	0	0	0.06
Intervillous lesions				
Massive histiocytic intervillositis	0	0	0	
Massive perivillous fibrin deposition	0	0	0	
Miscellaneous				
Chorangiosis	3 (13.6)	7 (17.9)	0	0.02 *
Chorangiomatosis	0	0	0	
Meconium effect	4 (18.2)	7 (17.9)	3 (9.7)	0.61
Villous immaturity	0	0	2 (6.5)	0.17
Villous edema	1 (4.5)	3 (7.7)	5 (16.1)	0.44

Data are presented as *n* (%). CVUE = chronic villitis of unknown etiology. * Represents statistically significant differences at *p* ≤ 0.05.
